# Social environment and services at the Special Immunobiological Reference Centers: epidemiological study, Minas Gerais, 2022-2024

**DOI:** 10.1590/S2237-96222026v35e20250092.en

**Published:** 2026-02-06

**Authors:** Thaís Moreira Oliveira, Ana Catarina de Melo Araújo, Giovanna Araújo Teixeira da Costa, Larissa Pereira Gomes, Tatianne Márcia Perdigão de Carvalho Alcantara, Natatia Santana Carvalho, Josianne Dias Gusmão, Mônica Levi, Ana Paula Neves Burian, Marcela Lencine Ferraz, Thales Philipe Rodrigues da Silva, Fernanda Penido Matozinhos

**Affiliations:** 1 Universidade Federal de Minas Gerais, Escola de Enfermagem, Belo Horizonte, MG, Brazil; 2 Ministério da Saúde, Brasília, DF, Brazil; 3 Prefeitura Municipal de Belo Horizonte, Belo Horizonte, MG, Brazil; 4 Secretaria de Estado de Saúde de Minas Gerais, Belo Horizonte, MG, Brazil; 5 Sociedade Brasileira de Imunização, São Paulo, SP, Brazil; 6 Secretaria de Estado de Saúde do Espírito Santo, Vitória, ES, Brazil

**Keywords:** Immunization Programs, Health Equity, Vaccines, Social Environment, Epidemiology, Programas de Inmunización, Equidad en Salud, Vacunas, Medio Social, Epidemiología

## Abstract

**Objective:**

To analyze individual, socioeconomic, demographic, and coverage factors of non-face-to-face services evaluated by the Special Immunobiological Reference Centers (*Centro de Referência para Imunobiológicos Especiais*, CRIE) of Minas Gerais, Brazil.

**Methods:**

Epidemiological study with a mixed design, using data from requests for non-face-to-face services received by the CRIE of Minas Gerais between 2022 and 2024. Categorical variables were described by absolute and relative frequencies (%), while continuous variables were described by median and interquartile range. Logistic models were used to assess the associations related to service provision, with the calculation of odds ratios (OR) and 95% confidence intervals (95%CI). Sociodemographic data of the municipalities were considered as independent variables.

**Results:**

A total of 9,158 requests were analyzed, with 51.8% of the requests coming from female individuals, and a median age of 51 years. Immunobiological agents were prescribed to 89.6% of individuals, with the 23-valent pneumococcal vaccine being the most frequently indicated (24.8%). The Regional Health Management Department of Coronel Fabriciano, Minas Gerais, showed the highest prevalence of referrals (n=1,232/13.4%), followed by the Regional Health Management Department of Ponte Nova (n=1,174/12.8%). Municipalities with a higher number of families earning up to half the minimum wage showed a lower likelihood of prescription (OR 0.99). Conversely, a higher proportion of the poor population registered in the Single Registry for Social Programs (*Cadastro Único*, CadÚnico) (OR 1.01) and a higher urbanization rate (OR 1.01) were associated with a greater likelihood of receiving a prescription.

**Conclusion:**

A potential association was found between social determinants of health and the non-face-to-face services evaluated by the CRIE, raising the discussion about the need to ensure equitable access to healthcare.

## Resumen

**Objetivo:**

Analizar los factores individuales, socioeconómicos, demográficos y de cobertura de las atenciones no presenciales evaluadas por el Centro de Referencia de Inmunobiológicos Especiales (*Centro de Referência para Imunobiológicos Especiais*, CRIE) de Minas Gerais, Brasil.

**Métodos:**

Estudio epidemiológico de diseño mixto, utilizando datos de las solicitudes de atenciones no presenciales recibidas por el CRIE de Minas Gerais entre 2022 y 2024. Las variables categóricas se describieron mediante frecuencia absoluta y relativa (%), mientras que las variables continuas se describieron mediante mediana e intervalo intercuartílico. Se aplicaron modelos logísticos para evaluar las asociaciones relacionadas con la atención, calculando razones de momios (*odds ratio*, OR) e intervalos de confianza del 95% (IC95%). Como variables independientes se consideraron datos sociodemográficos de los municipios.

**Resultados:**

Se analizaron 9.158 solicitudes, de las cuales el 51,8% correspondieron al sexo femenino, con una mediana de edad de 51 años. Se prescribieron inmunobiológicos al 89,6% de los individuos, siendo la vacuna neumocócica 23-valente la más indicada (24,8%). La Gerencia Regional de Salud de Coronel Fabriciano presentó la mayor prevalencia de derivaciones (n=1.232; 13,4%), seguida de la regional de Ponte Nova (n=1.174; 12,8%). Los municipios con mayor número de familias con ingresos de hasta medio salario mínimo presentaron menor probabilidad de prescripción (OR 0,99), mientras que una mayor proporción de población pobre registrada en el CadÚnico (*Cadastro Único*, CadÚnico) (OR 1,01) y una mayor tasa de urbanización (OR 1,01) se asociaron a una mayor probabilidad de prescripción.

**Conclusión:**

Se identificó un potencial de asociación entre los determinantes sociales de la salud y las atenciones no presenciales evaluadas por el CRIE, lo que plantea la discusión sobre la necesidad de garantizar la equidad en el acceso a los servicios de salud.

**Ethical aspects**

This research respected ethical principles, having obtained the following approval data:

Research ethics committee: Universidade Federal de Minas Gerais

Opinion number: 6,739,290

Approval date: 2/4/2024

Certificate of submission for ethical appraisal: 78079324,2,0000,5149

Informed consent form: Exempt.

## Introduction 

In 1973, the National Immunization Program (*Programa Nacional de Imunizações*, PNI) was established in Brazil to ensure the free distribution of various immunizing agents to the population, thereby contributing to the prevention of vaccine-preventable diseases through vaccination activities (1). In this context, the 1988 Constitution established the Brazilian National Health System (*Sistema Único de Saúde*, SUS), intending to guarantee the population universal, equitable, and comprehensive access to goods and services that promote health and well-being (2). 

In 1993, the PNI was expanded through the creation of the Special Immunobiological Reference Centers (*Centro de Referência para Imunobiológicos Especiais*, CRIE), public centers with specific infrastructure and logistics that provide special immunobiological agents (vaccines and immunoglobulins) for individuals with specific clinical conditions, such as chronic diseases or higher risk of infection (3). These centers provide special immunizing agents free of charge to promote equity in access to those that are not available in conventional vaccination schedules but are essential for population groups with special needs (1,3).

**Figure 1:**
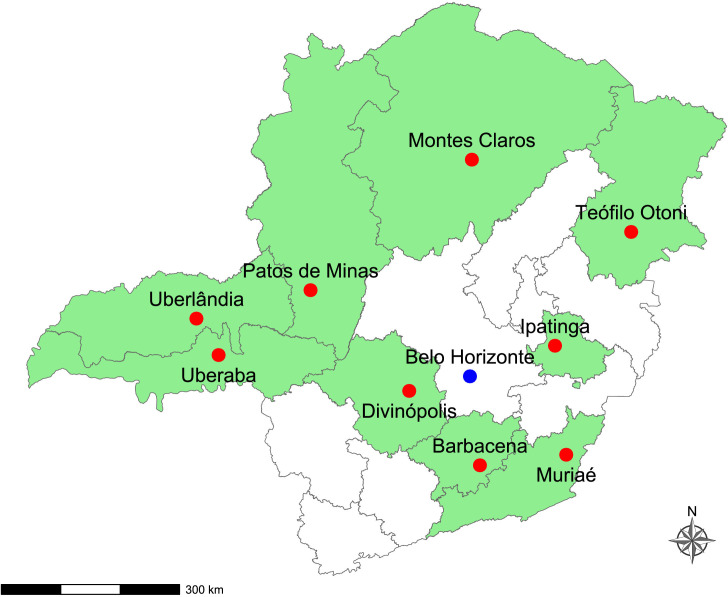
Location of physical Special Immunobiological Reference Centers units in the state. Minas Gerais, 2025

In Minas Gerais, the CRIE of Minas Gerais, established in 1994, plays a strategic role not only as a physical unit in Belo Horizonte, with in-person services, but also as a Virtual CRIE, serving as a coordinating reference for other municipalities in the state that do not have CRIE units in their macroregions (4). By the end of 2025, the state will have a total of ten CRIE units, located in Belo Horizonte, Uberlândia, Juiz de Fora, Divinópolis, Patos de Minas, Montes Claros, Ipatinga, Barbacena, Teófilo Otoni, and Muriaé (Figure 1). 

Although SUS is grounded in universality and comprehensiveness, regional inequalities persist, especially in the North and Northeast regions of the country, regarding access to essential health services (5). In addition to inequalities in access to healthcare there are disparities related to the social environment, which manifest in various dimensions, including income and wealth, gender, race, and place of residence (6,7). Despite strategies undertaken to minimize the impact of these differences (2,6), inequities in access to and use of health services persist in Brazil (8,9). Gaps in access to vaccines, which compromise the achievement of immunization targets, are also disparities found both within and between regions (10). SUS has expanded access to health services, accompanied by a reduction in health inequalities within the population, although access to specialized care remains a challenge (11).

This study aimed to analyze individual, socioeconomic, demographic, and coverage factors of non-face-to-face services evaluated by the CRIE of Minas Gerais and to identify the determinants that may influence access to special immunobiological agents. The research aims to enhance understanding of the factors that may affect access to this resource, especially in contexts of social inequality, and reinforces the relevance of the CRIE of Minas Gerais in promoting health equity in the state. 

## Methods 

### Study design

This was an epidemiological study with a mixed design, using data on requests for virtual services from the CRIE in Belo Horizonte, Minas Gerais, Brazil, from September 2022 to July 2024.

### Setting 

Until 2013, requests for Virtual CRIE services at the CRIE of Minas Gerais were recorded through the Information System of the Special Immunobiological Reference Centers (*Sistema de Informação dos Centros de Referência para Imunobiológicos Especiais*, SI-CRIE), a computerized platform designed to manage user data and special immunobiological agents (1,3). With the increase in demand and volume of requests, the SI-CRIE became insufficient to meet the service’s recording needs effectively. Given this limitation and based on more recent guidelines, records of Virtual CRIE services began to be made through Google Forms, with automatic storage in Google Sheets, which facilited organization and optimized information management. This new format allows for detailed user registration, scheduling of vaccination schemes, and monitoring of released vaccines, ensuring greater control and efficiency of information by the CRIE of Minas Gerais.

As a Virtual CRIE, the CRIE of Minas Gerais receives requests for special immunobiological agents sent by the Regional Health Centers, analyzes them, and, if the indication is confirmed, authorizes and organizes the logistics so that the immunobiological agent is provided in the state’s macroregions that do not have a regional CRIE (1,3). This strategy is also adopted by other CRIE units in the network, optimizing operational flows in the health macroregions and expanding the response capacity to requests received by the CRIE (4). The 28 Regional Health Management Offices complete the immunobiological request forms, prepared according to the criteria of the most recent CRIE manual, and submit them virtually to the CRIE of Minas Gerais.

All requests received and evaluated by the CRIE of Minas Gerais team are recorded in the Google Forms spreadsheet. When the user indicates that they wish to receive the immunobiological agent, the request is returned to the responsible regional management office, which then forwards the demand to the Municipal Health Department. The user’s vaccination takes place at the vaccination center or in primary health care, depending on the municipality’s context. The service administering the vaccine is responsible for recording it in the current immunization systems.

### Participants 

The participants of this study were the users of the CRIE of Minas Gerais with requests recorded in the Virtual CRIE spreadsheets between September 2022 and July 2024. Referrals in which the user’s municipality of residence could not be correctly identified were excluded. Some variables contained incorrect or invalid records, reducing the number of observations depending on the variable analyzed. For the variable “indication for the immunobiological agent,” categorization was performed according to the indications of the current CRIE manual (3). The “other indications” category encompassed those with an occurrence rate of less than 1.0% in the study sample.

### Variables 

The variables used in this study included the users’ sociodemographic profile, data from the referring health service, and specific information from the requests for immunobiological agents. As independent variables, the municipalities’ sociodemographic data were adopted through the 2021 Minas Gerais Social Responsibility Index (*índice mineiro de responsabilidade social*, IMRS) (12,13), from the Foundation João Pinheiro (*Fundação João Pinheiro*, FJP), and 2024 indices from the Brazilian Institute of Geography and Statistics (*Instituto Brasileiro de Geografia e Estatística*, IBGE), available on public domain websites (https://imrs.fjp.mg.gov.br/Consultas and https://www.ibge.gov.br/estatisticas/sociais/populacao/9103-estimativas-de-populacao.html, respectively).

### Data sources and measurement

The data were obtained by extracting information recorded in the spreadsheets derived from the request forms for special immunobiological agents received by the CRIE of Minas Gerais for Virtual CRIE services. 

### Bias control

To avoid potential sources of bias, a consistency check of the records was conducted after receiving the spreadsheet, which identified incorrect or missing data in the “municipality” variable, rendering their inclusion in the study impossible. Thus, 742 records were excluded from the analyses in this study.

### Study size

The study included all requests recorded between September 2022 and July 2024, encompassing the entirety of non-face-to-face services evaluated by the CRIE of Minas Gerais. 

### Quantitative variables

The sociodemographic profile variables included “sex” (male/female) and “age” (both numerical and categorical). To characterize the referring service, the Regional Health Management Departments were used (28 categories). Information from the immunobiological requests was described by the indications/reasons for referral to CRIE (15 categories) and by the immunobiological agents prescribed (19 categories). The municipalities’ sociodemographic variables (families registered with up to half the minimum wage; percentage of poor population in CadÚnico; proportion of the population covered by the Family Health Strategy; municipality urbanization rate; and estimated municipality population) (12,13) are presented in Table 1.

**Table 1:** Social environment variables used in the study’s logistic regression, based on the individual’s municipality of residence. Belo Horizonte, 2022–2024

Variables	Concept
Families registered with up to half a minimum wage	Number of families registered with a per capita income of up to half a minimum wage, with up to two years from the date of its inclusion or last cadastral update.
Percentage of the poor population in CadÚnico	Proportion of the population registered in CadÚnico classified as poor, according to government-established income criteria.
Proportion of the population covered by the Family Health Strategy	Percentage of the population covered by the Family Health Strategy in a given geographic are, in the year considered.
Urbanization rate of the municipality	Percentage of the population living in urban areas compared to the total population of the municipality based on data from the Brazilian Institute of Geography and Statistics (IBGE).
Estimated population of the municipality	IBGE, Directorate of Surveys, Division of Population and Social Indicators: estimates of the resident population in Brazil and its states, with reference date of July 1, 2024.

### Statistical methods

Data were analyzed using Stata statistical software, version 17.0. Categorical variables were described by absolute and relative frequencies, and continuous variables, after checking for data skewness, were presented as median and interquartile range (IQR). To estimate the magnitude of associations between immunobiological prescription, users’ sociodemographic profile, and municipality of origin, crude and adjusted odds ratios (OR) with their respective 95% confidence intervals were calculated using logistic regression models.

Choropleth maps were created using QGIS, version 3.40.0, to represent the spatial distribution of referrals made during the study period.

## Results 

**Figure 2:**
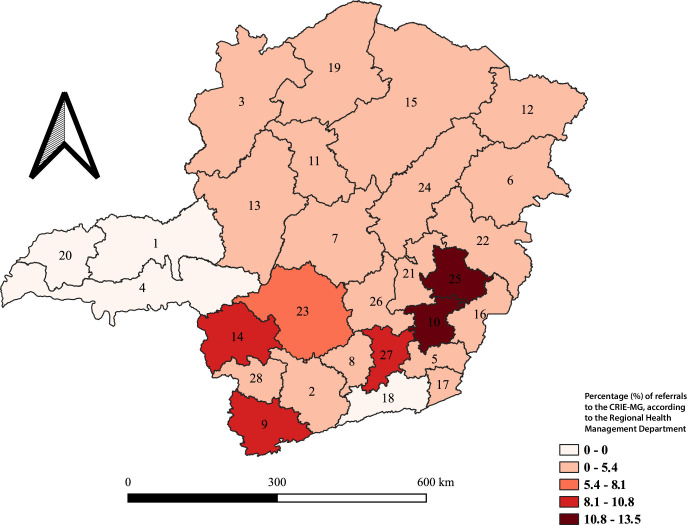
Regional Health Management Departments that referred special immunobiological agents request forms for non-face-to-face care at the Special Immunobiological Reference Centers. Minas Gerais, 2022–2024 (n=9,158)

During the study period, the CRIE of Minas Gerais received 9,900 requests for immunobiological agents virtually. After applying the exclusion criteria, 9,158 requests were included in the analysis. The majority of individuals requesting special immunobiological agents were female (51.8%). The median age was 51 years (IQR 26–77), with predominance in the adult age group, which accounted for 3,681 (40.2%) of cases (Table 2). The most prevalent indications for receiving immunobiological agents were people living with HIV/aids (16.2%) and chronic lung disease, pulmonary diseases, or asthma (13.1%) (Table 2). Among the data related to the referring health service, the Regional Health Management Department of Coronel Fabriciano stood out, being responsible for 13.4% of referrals (Figure 2).

**Table 2:** Sex, age group, and indications for special immunobiological agents among users referred for remote care at the Special Immunobiological Reference Centers of Minas Gerais. Belo Horizonte, 2022–2024

Sociodemographic variables^a^	n (%)
Sex	
Female	4,736 (51.8)
Male	4,402 (48.2)
**Age group** (years)	
0–9	1,699 (18.6)
10–18	210 (2.3)
19–59	3,681 (40.2)
>60	3,553 (38.9)
**Indications/reasons for referrals** (,158)	
People living with HIV/aids	1,481 (16.2)
Chronic lung disease, pulmonary diseases, asthma	1,197 (13.1)
Immunocompromised/immunodeficiency due to therapeutic immunosuppression/acquired and congenital immunodeficiency	944 (10.3)
Neoplasms	787 (8.6)
No criterion/no underlying disease/awaiting documentation	783 (8.5)
Diabetes	758 (8.3)
Susceptible newborn – prematurity	537 (5.9)
Chronic cardiovascular disease	531 (5.8)
Anatomic or functional asplenia, hemoglobinopathies, storage diseases, and other conditions associated with splenic dysfunction	452 (4.9)
Transplants (solid organs/bone marrow/donors)	436 (4.8)
Chronic kidney disease/nephrotic syndrome	352 (3.8)
Disabling chronic neurological disease/ chronic seizure disorder/encephalopathies	336 (3.6)
Other indications^b^	210 (2.3)
Liver diseases	200 (2.2)
Genetic syndromes	154 (1.7)

^a^Totals by variable may vary due to incomplete records. ^b^Other indications: previous adverse event/others/cochlear implant/CSF leak and ventriculoperitoneal shunt/post-exposure prophylaxis for risk/sexual abuse/household contacts of HBV carriers/newborn of HBsAg-positive mother/rheumatologic diseases/severe chronic dermatologic diseases/health Professional/percutaneous accident/mucosal contact with index case HBsAg (+) or high risk/pregnant people/purpura (asplenia/immunosuppression)/sexual contacts/storage diseases.

Of the 9,158 records, prescription and release of immunobiological agents were completed for 8,211 individuals (89.6%). It should be noted that the same individual may have had indications for more than one immunobiological agent, resulting in a total of 28,588 prescribed doses. The most frequently indicated vaccines were: 23-valent pneumococcal (,098; 24.8%), *Haemophilus influenzae type b* (,019; 13.6%), and hepatitis A (,885; 14.1%) (Table 3).

**Table 3:** Immunobiological agents prescribed for users referred for non-face-to-face care at the Special Immunobiological Reference Centers of Minas Gerais. Belo Horizonte, 2022–2024 (n=28,588)

Immunobiological agents prescribed	n (%)
23-valent pneumococcus	7,098 (24.8)
Haemophilus influenzae type B (Hib)	4,019 (14.1)
Hepatitis A	3,885 (13.6)
Meningococcal C conjugate vaccine	3,106 (10.9)
13-valent pneumococcal conjugate vaccine (PCV13)	2,684 (9.4)
Influenza	1,708 (6.0)
HPV4^a^	1,266 (4.4)
Meningococcal conjugate vaccine (ACWY)	1,041 (3.6)
Hepatitis B	998 (3.5)
DTaP^b^	937 (3.3)
Hexavalent acellular vaccine (DTaP-IPV-Hib-HepB)^c^	542 (1.9)
dT^d^	465 (1.6)
Varicella	447 (1.6)
Poliovirus inactivated	182 (0.6)
MMR	116 (0.4)
Yellow fever	66 (0.2)
Hepatitis B Immune Globulin (HBIG)	23 (0.1)
Tetanus Immune Globulin (TIG)	3 (0.0)
Rabies Immune Globulin (RIG)	2 (0.0)
Total	**28,588** (100.0)

^a^Human papillomavirus vaccine types 6, 11, 16, and 18 (recombinant); ^b^Adsorbed diphtheria, tetanus, and (acellular) pertussis vaccine; ^c^Diphtheria, tetanus, pertussis, Haemophilus influenzae type b, poliomyelitis, and hepatitis B; ^d^Diphtheria and tetanus vaccine.

To estimate the magnitude of the association between individual variables and social environment variables with immunobiological prescription, the adjusted analysis showed that higher age was associated with a greater likelihood of prescription. Female sex also showed a higher likelihood of prescription compared to male, with statistical significance. Regarding the independent variables related to the social environment of the municipalities of residence, it was observed that the greater the number of families with incomes up to half the minimum wage, the lower the likelihood of prescription of immunobiological agents. On the other hand, municipalities with higher percentages of poor populations registered in CadÚnico and higher urbanization rates showed a greater likelihood of prescription (p-value<0.05) (Table 4).

**Table 4:** Adjusted odds ratios (OR) and adjusted 95% confidence intervals (95%CI) for the prescription of immunobiological, agents according to individual and social environment variables. Belo Horizonte, 2022–2024

Variables	Adjusted OR (95%CI)	p-value
**Individual variables**		
**Age** (years)	1.02 (1.01; 1.02)	<0.001
Sex		<0.001
Female	reference	
Male	1.57 (1.36; 1.80)	
**Social environment variables**		
Number of families with per capita income up to half a minimum wage	0.99 (0.99; 1.00)	0.050
Percentage of the population classified as poor according to CadÚnico	1.01 (1.00; 1.03)	0.004
Proportion of the population covered by the Family Health Strategy	0.99 (0.99; 1.00)	0.533
Urbanization rate	1.01 (1.00; 1.02)	0.026
Estimated population	1.00 (0.99; 1.00)	0.255

## Discussion 

The results of this study demonstrated a possible association between the prescription of immunobiological agents in non-face-to-face services, as evaluated by the CRIE of Minas Gerais, and individual and health-related environmental variables. This finding underscores the importance of ensuring equitable access to health services as a crucial commitment to achieving the sustainable development goals.

In this research, the majority of individuals requesting special immunobiological agents were female, with a median age of 51 years. This analysis reflects current demographic and epidemiological trends, in which population aging, combined with higher life expectancy among women, underscores the need for public policies that promote healthy aging and quality of life across all stages of life (14). In Brazil, in 2021, vaccine-preventable diseases were the second leading cause of death among those preventable through SUS interventions for women aged 5 to 74 years (15).

An analysis covering the period from 1998 to 2008 indicated that the difference in access to and use of health services between genders remained significant, with men having access seven percentage points lower than women (16). In 2023, data from the Ministry of Health indicated an increase in individual consultations for men in Primary Health Care compared to 2022. However, men still represented only 33.4% of services at the national level (11). Among the entities responsible for referring non-face-to-face services to the CRIE of Minas Gerais, the Regional Health Management Departments of Coronel Fabriciano and Ponte Nova stood out, located in the Vale do Aço and Zona da Mata regions of Minas Gerais, respectively. This prevalence can be attributed to the fact that, until 2024, these regions lacked a physical CRIE unit, which contributed to the high demand for services and justified the recent establishment of a center in Ipatinga, administered by the Regional Health Management Department of Coronel Fabriciano (4).

Requests for Virtual CRIE services made by users from municipalities belonging to the Uberaba Regional Health Management Departments are directed to the Uberaba Macrorregional CRIE. The Uberlândia Macrorregional CRIE, in turn, is responsible for municipalities in the Uberlândia and Ituiutaba Regional Health Management Departments. The CRIE in Juiz de Fora serves the municipalities of its respective Regional Health Management Department. In this way, these departments may not submit virtual service requests to the CRIE of Minas Gerais, which may explain the absence of such referrals during the period analyzed in this study.

As part of this organization, since the end of 2022, the State Health Department has been mobilizing to structure workflows and develop capacities aimed at decentralizing CRIE services in Minas Gerais. The pursuit of reducing inequalities in access to and quality of services through equity and decentralization is a fundamental pillar to ensure that health care meets the diverse needs of the population (17,18). 

Almost all Virtual CRIE requests evaluated by the CRIE of Minas Gerais resulted in the prescription of special immunobiological agents for the referred individuals. The accuracy of the professionals responsible for the referrals highlights the importance of technical knowledge regarding the target groups and their respective recommendations. Indications, differentiated vaccination schedules, pre-established criteria, and the addresses of CRIE units are available in the CRIE Manual (3).

Mastery of workflows, indications, norms, and procedures required for referring high-risk individuals underscores the discussion on these users’ access to health services (5–10). It was not possible to verify whether the prescribed immunobiological agents were actually administered, nor the time interval between the request, approval, and application of the doses. This limitation stems from the lack of integration between the analyzed records and national health information systems, such as SI-PNI and the Brazilian National Health Electronic System (*Sistema Único de Saúde Eletrônico*, e-SUS), where the receipt and administration of immunobiological agents are registered.

Among the indications/reasons for referrals to vaccination at CRIE, the most notable were conditions affecting people living with HIV/aids and chronic lung disease, other pulmonary diseases, and asthma. In this study, the 23-valent pneumococcal vaccine was the most frequently prescribed. Community-acquired pneumonia was identified as the third most prevalent coinfection among people living with HIV in a longitudinal study (19). These findings reinforce that HIV infection makes individuals more vulnerable to infections compared to the general population (3). 

A meta-analysis demonstrated a reduction in pediatric pneumonia cases caused by *Haemophilus influenzae* type B (Hib) following the approval of the Hib conjugate vaccine in China, indicating that the expanded use of this vaccine contributed to a decrease in the pathogen’s impact among the causes of childhood pneumonia in the country (20). The second most prescribed vaccine was for hepatitis A. The incidence of this infection in low-income populations occurs at younger ages due to inadequate access to basic sanitation. Conversely, in regions with better sanitary conditions, a higher incidence is observed among adolescents and adults (3). 

CRIE may also recommend vaccines not prescribed by the referring physician, emphasizing the importance of a technical team composed of both a physician and a nurse in the Virtual CRIE (3,4). After reviewing the requests, these professionals can expand access to the vaccines provided by the service. Thus, non-face-to-face services evaluated by the CRIE of Minas Gerais may also be influenced by contextual factors, particularly of a social nature.

This study aimed to investigate the existence of inequalities or implicit social patterns in the prescription of special immunobiological agents, despite these prescriptions being based exclusively on clinical criteria. The results demonstrated that increased age was associated with a higher likelihood of being prescribed immunobiological agents, and women had a higher probability of prescription compared to men. This heterogeneity between men and women, as well as between younger and older individuals, was also observed in a study on COVID-19 vaccination coverage in Brazil, which found that older populations were prioritized for vaccination and that, across all age groups, vaccine coverage among women remained higher than that observed among men over time (21).

The model identified that individuals residing in municipalities with higher urbanization rates had a greater likelihood of receiving prescriptions for immunobiological agents. A study conducted in Ethiopia comparing fully vaccinated children in urban and rural areas found a higher prevalence of complete vaccination among urban residents (22).

In this study, it was also observed that the number of families with income up to half the minimum wage was associated with a lower likelihood of prescription. In contrast, a higher percentage of the poor population registered in the CadÚnico was related to a higher probability of prescription. Therefore, two socioeconomic indicators showed a possible association with the likelihood of an individual receiving immunobiological agents. During the pandemic, income also influenced the continuation of the vaccination schedule: residents of municipalities with lower per capita income were less likely to receive the second dose of the COVID-19 vaccine (21).

A global analysis synthesized evidence on socioeconomic inequalities in routine vaccine acceptance, revealing a positive association between higher socioeconomic status and adherence to vaccination in most studies (23). On the other hand, a survey on vaccination coverage and hesitancy in Brazilian capitals revealed lower vaccine uptake among the more affluent strata compared to the more vulnerable (24). Therefore, equitable vaccine distribution at both national and international levels is crucial to ensure universal access to immunization and reduce health inequalities (7–9,^25^,^26^).

A study in sub-Saharan Africa found that children from higher-income families had higher vaccination coverage rates compared to those from poorer families (27). In addition to economic conditions, factors related to physical access to health services, such as proximity to Primary Health Care Centers, opening hours, and temporary vaccine shortages, also significantly influenced adherence (27). Access to information, particularly through health professional recommendations, also has a demonstrated impact on the decision to vaccinate oneself or dependents (27).

In 2025, to expand access and optimize the services provided by CRIE, the Ministry of Health established, through an ordinance, the creation of the Network of Immunobiological Agents for People in Special Situations (*Rede de Imunobiológicos para Pessoas em Situações Especiais*, RIE). This new strategy does not replace CRIE, which remains responsible for validating indications, administering special vaccines, and acting as territorial references in the Bipartite Intermanagerial Comission (*Comissões Intergestores Bipartite*, CIB). The main innovation introduced by the RIE is the expansion of service coverage, allowing Primary Health Care, through Health Centers, also to vaccinate individuals with special conditions, thereby fostering integration across levels of care and promoting greater equity in access to immunobiological agents (28).

Finally, the results of this study are believed to provide important evidence for the improvement and operationalization of CRIE by highlighting opportunities in the process of accessing special immunobiological agents available to populations in special situations, considering their social context. In practice, this information enables the development of planning, monitoring, and evaluation actions aimed at optimizing equitable access to special immunobiological agents, as well as contributing to the reorganization of workflows and the expansion of vaccination in vulnerable populations.

This study has the limitation of using secondary data, which may be subject to incorrect or incomplete entries. Additionally, since the data were anonymized, it was not possible to identify the same individual across different records, a criterion necessary to ensure the independence of the explanatory variables used in the regression model. Another limiting factor was the lack of information, which prevented us from distinguishing whether the referring health service was public or private, and could have enriched the analysis of social context variables. It is also important to note that it was not possible to confirm either the administration or the time elapsed between the request, release, and application of the immunobiological agent, due to the lack of integration between the registration systems. Nevertheless, the study employed a rigorous methodology that allowed for the identification of the random pattern of missing data and the conduct of sensitivity analyses to ensure the reliability of the results.

A potential association was found between environmental health determinants and the non-face-to-face services evaluated by the CRIE of Minas Gerais. Identifying the social environment profile of these services in Minas Gerais raises discussion about the need to guarantee equity in access to healthcare. Expanding research and data that measure the impact of equitable actions enables assessment of progress and the proposal of more effective interventions to reduce inequalities. The expansion of research and data that measure the impact of equitable actions allows us to evaluate progress and propose more effective interventions to combat inequalities.

## Data Availability

The data used in this study cannot be made publicly available in full due to ethical restrictions established by the Research Ethics Committee that approved the study, under Certificate of Submission for Ethical Appraisal No. 78079324,2,0000,5149 and Opinion No. 6,739,290. In this way, the duly anonymized data were deposited in the SciELO Data repository, and the ethical requirements regarding confidentiality and data privacy were strictly followed.

## References

[1] Brasil (2024 ). Manual de normas e procedimentos para vacinação.

[2] Machado CV (2024). Democracy, citizenship and health in Brazil: challenges to strengthening the Unified Health System (SUS). Ciênc. Saúde Colet.

[3] Brasil (2023 ). Manual dos Centros de Referência para Imunobiológicos Especiais.

[4] Minas Gerais (2024 ). Nota Técnica nº 12 SES/SUBVS/SVE/DVDTI/2024: descentralização dos Centros de Referência para Imunobiológicos Especiais (CRIE) e da vigilância dos Eventos Supostamente Atribuíveis a Vacinação ou Imunização (ESAVI).

[5] Pitombeira DF, de Oliveira LC (2020). Poverty and social inequality: tensions between rights and austerity and its implications for primary healthcare. Ciênc. Saúde Colet.

[6] Souza LEPF, Barros RD, Barreto ML, Katikireddi SV, Hone TV, Paes Sousa R (2019). The potential impact of austerity on attainment of the Sustainable Development Goals in Brazil. BMJ Glob Health.

[7] Guan A, Shariff-Marco S, Henry K, Lin K, Meltzer D, Canchola AJ (2025). Latino enclaves and healthcare accessibility: an ecologic study Across Five States. J Gen Intern Med.

[8] Palmeira NC, Moro JP, Getulino FA, Vieira YP, Soares AO, Saes MO (2019). Análise do acesso a serviços de saúde no Brasil segundo perfil sociodemográfico: Pesquisa Nacional de Saúde, 2019. Epidemiol. Serv. Saude.

[9] Galvão ALM, Oliveira E, Germani ACCG, Luiz OC (2021). Determinantes estruturais da saúde, raça, gênero e classe social: uma revisão de escopo. Saúde Soc.

[10] Lima JC, Garcia EM, Oliveira SMVL, Araújo WN, Lopes EMF, Teles SA (2017). Vaccine coverage by social strata in state capitals in the Brazilian Midwest region: a household survey of children born in 2017 and 2018. Epidemiol Serv Saude.

[11] Brasil (2023). Relatório de Gestão 2023.

[12] Brasil (2022 ). Famílias cadastradas com até meio salário mínimo atualizadas.

[13] Fundação Oswaldo Cruz (2020 ). Projeto de Avaliação do Desempenho do Sistema de Saúde (PROADESS) - Avaliação do Desempenho do Sistema de Saúde [Internet].

[14] Buss PM, Araújo Hartz ZM, Pinto LF, Rocha CMF (2020). Health promotion and quality of life: a historical perspective of the last two 40 years (1980-2020). Ciênc. Saúde Colet.

[15] Brasil (2023 ). Saúde da mulher brasileira: uma perspectiva integrada entre vigilância e atenção à saúde.

[16] Arruda NM, Maia AG, Alves LC (1998). Inequality in access to health services between urban and rural areas in Brazil: A disaggregation of factors from 1998 to 2008. Cad. Saude Publica.

[17] Brasil (2001 ). Regionalização da assistência à saúde: aprofundando a descentralização com equidade no acesso: Norma Operacional da Assistência à Saúde: NOAS-SUS 01/01 e Portaria MS/GM n.º 95.

[18] Ribeiro JM, Moreira MR, Ouverney AM, Silva CMFP (2017). Health policies and federative gaps in Brazil: An analysis of regional capacity of services delivery. Ciênc. Saúde Colet.

[19] Valladales-Restrepo LF, Oyuela-Gutiérrez MC, Díaz-Arteaga C, Torres-Campo MA, Rengifo-Montes A, Erazo-De Los Ríos AS (2024). Coinfections and In-Hospital Mortality in a Group of Patients With HIV/AIDS: A Longitudinal Study. Inquiry.

[20] Abavisani M, Keikha M, Karbalaei M (2024). First global report about the prevalence of multi-drug resistant Haemophilus influenzae: a systematic review and meta-analysis. BMC Infect Dis.

[21] Li SL, Prete CA, Zarebski AE, de Souza Santos AA, Sabino EC, Nascimento VH (2024). The Brazilian COVID-19 vaccination campaign: a modelling analysis of sociodemographic factors on uptake. BMJ Open.

[22] Asmare G, Madalicho M, Sorsa A (2022). Disparities in full immunization coverage among urban and rural children aged 12-23 months in southwest Ethiopia: A comparative cross-sectional study. Hum Vaccin Immunother.

[23] Sacre A, Bambra C, Wildman JM, Thomson K, Bennett N, Sowden S (2023). Socioeconomic inequalities in vaccine uptake: A global umbrella review. PLoS One.

[24] Moraes JC, França AP, Guibu IA, Barata RB, Domingues CMAS, Teixeira MG (2023 ). Inquérito de cobertura vacinal nas capitais brasileiras, Distrito Federal e em 12 municípios do interior, em crianças nascidas em 2017-2018 e residentes nas áreas urbanas [Internet].

[25] United Nations Development Programme (2022 ). Vaccine equity: boosting speeds, accelerating equity [Internet].

[26] Domingues CMAS, Maranhão AGK, Teixeira AM, Fantinato FFS, Domingues RAS (2020). The Brazilian National Immunization Program: 46 years of achievements and challenges. Cad Saude Publica.

[27] Xavier SP, Mahoche M, Rondó PH, Silva AM, Flores-Ortiz R, Victor A (2024). Addressing inequalities in vaccination coverage among children aged 12 to 23 months in ten Sub-Saharan African countries: Insights from DHS and MIS Data (2017-2022).

[28] Brasil (2025). Portaria GM/MS nº 6.623, de 14 de fevereiro de 2025. Institui a Rede de Imunobiológicos para Pessoas com Situações Especiais - RIE. Diário Oficial da União.

