# Supplementary material for: Social environment and services at the Special Immunobiological Reference Centers: epidemiological study, Minas Gerais, 2022-2024
Source: Epidemiol Serv Saude. 2026 Feb 6;35:e20250092. doi: 10.1590/S2237-96222026v35e20250092.en (PMC12885467; doi:10.1590/S2237-96222026v35e20250092.en)
Supplement: Translation

# Translation: Ambiente social e atendimentos do Centro de Referência para Imunobiológicos Especiais: estudo epidemiológico, Minas Gerais, 2022-2024

**DOI:** 10.1590/S2237-96222026v35e20250092.pt

**Authors:** Thaís Moreira Oliveira, Ana Catarina de Melo Araújo, Giovanna Araújo Teixeira da Costa, Larissa Pereira Gomes, Tatianne Márcia Perdigão de Carvalho Alcantara, Natatia Santana Carvalho, Josianne Dias Gusmão, Mônica Levi, Ana Paula Neves Burian, Marcela Lencine Ferraz, Thales Philipe Rodrigues da Silva, Fernanda Penido Matozinhos

**Affiliations:** 1 Universidade Federal de Minas Gerais, Escola de Enfermagem, Belo Horizonte, MG, Brasil; 2 Ministério da Saúde, Brasília, DF, Brasil; 3 Prefeitura Municipal de Belo Horizonte, Belo Horizonte, MG, Brasil; 4 Secretaria de Estado de Saúde de Minas Gerais, Belo Horizonte, MG, Brasil; 5 Sociedade Brasileira de Imunização, São Paulo, SP, Brasil; 6 Secretaria de Estado de Saúde do Espírito Santo, Vitória, ES, Brasil

**Keywords:** Programas de Imunização, Equidade em Saúde, Vacinas, Meio Social, Epidemiologia

## Abstract

**Objetivo:**

Analisar fatores individuais, socioeconômicos, demográficos e de cobertura dos atendimentos não presenciais avaliados pelo Centro de Referência para Imunobiológicos Especiais (CRIE) de Minas Gerais, Brasil.

**Métodos:**

Estudo epidemiológico com delineamento misto, utilizando dados das solicitações de atendimentos não presenciais recebidas pelo CRIE de Minas Gerais entre 2022 e 2024. Variáveis categóricas foram descritas por frequências absoluta e relativa (%), enquanto as contínuas foram descritas por mediana e intervalo interquartílico. Modelos logísticos avaliaram as associações relacionadas ao atendimento, com cálculo de razão de chances (*odds ratio*, OR) e intervalos de confiança de 95% (IC95%). Como variáveis independentes, foram considerados dados sociodemográficos dos municípios.

**Resultados:**

Foram analisadas 9.158 solicitações, sendo 51,8% do sexo feminino, com mediana de idade de 51 anos. Imunobiológicos foram prescritos para 89,6% dos indivíduos, sendo a vacina pneumocócica 23-valente a mais indicada (24,8%). A gerência regional de saúde de Coronel Fabriciano, Minas Gerais, apresentou a maior prevalência de encaminhamentos (n=1.232/13,4%), seguida da regional de Ponte Nova (n=1.174/12,8%). Municípios com maior número de famílias com renda de até meio salário mínimo apresentaram menor chance de prescrição (OR 0,99). Já a maior proporção de população pobre cadastrada no CadÚnico (OR 1,01) e a maior taxa de urbanização (OR 1,01) estiveram associadas a uma maior chance de prescrição.

**Conclusão:**

Houve potencial de associação entre os determinantes sociais da saúde e os atendimentos não presenciais avaliados pelo CRIE, o que suscita a discussão acerca da necessidade de garantia da equidade no acesso à saúde.

**Aspectos éticos**

Esta pesquisa respeitou os princípios éticos, obtendo os seguintes dados de aprovação:

Comitê de ética em pesquisa: Universidade Federal de Minas Gerais

Número do parecer: 6.739.290

Data de aprovação: 2/4/2024

Certificado de apresentação de apreciação ética: 78079324.2.0000.5149

Registro do consentimento livre e esclarecido: Não se aplica.

## Introdução 

Em 1973, foi instituído, no Brasil, o Programa Nacional de Imunizações (PNI), com o objetivo de garantir a distribuição gratuita de diferentes tipos de imunizantes para a população, contribuindo para a prevenção de doenças preveníveis por ações de vacinação por meio de ações de vacinação (1). Nesse contexto, a Constituição de 1988 instituiu o Sistema Único de Saúde (SUS), com o objetivo de garantir à população o acesso universal, equitativo e integral a bens e a serviços que promovam a saúde e o bem-estar. (2). 

Em 1993, o PNI foi ampliado através da criação dos Centros de Referência para Imunobiológicos Especiais (CRIE), centros públicos com infraestrutura e logística específicas que oferecem imunobiológicos especiais (vacinas e imunoglobulinas) para indivíduos com condições clínicas específicas, como doenças crônicas ou risco maior de infecção (3). Esses centros ofertam gratuitamente imunizantes especiais para promover a equidade no acesso àqueles que não estão disponíveis nos calendários vacinais convencionais, mas que são indispensáveis para grupos populacionais com necessidades especiais (1,3).

Em Minas Gerais, o CRIE Minas Gerais, implantado em 1994, desempenha um papel estratégico, não apenas como unidade física em Belo Horizonte, com atendimentos presenciais, mas também como CRIE Virtual, atuando como referência matriciadora para outros municípios do estado que não possuem unidades do CRIE em suas macrorregionais (4). Até o final de 2025, o estado contará com um total de dez unidades do CRIE, localizadas em Belo Horizonte, Uberlândia, Juiz de Fora, Divinópolis, Patos de Minas, Montes Claros, Ipatinga, Barbacena, Teófilo Otoni e Muriaé (Figura 1).

**Figura 1:** Localização dos Centros de Referência para Imunobiológicos Especiais físicos no estado. Minas Gerais, 2025

Embora o SUS tenha como premissa a universalidade e a integralidade, persistem desigualdades regionais, com destaque para as regiões Norte e Nordeste do país, no acesso a serviços essenciais de saúde (5). Além das desigualdades no acesso à saúde, existem disparidades relacionadas ao ambiente social que se manifestam em diferentes dimensões, como renda e riqueza, gênero, raça e local de moradia (6,7). Apesar das estratégias realizadas para minimizar o impacto dessas diferenças (2,6), persistem as iniquidades no acesso e na utilização dos serviços de saúde no Brasil (8,9). Lacunas no acesso às vacinas, que comprometem o sucesso em atingir as metas de imunização, também são disparidades encontradas tanto intra quanto inter-regionalmente (10). O SUS expandiu o acesso aos serviços de saúde, acompanhado da redução das desigualdades em saúde da população, embora o acesso a cuidados especializados ainda represente um desafio (11).

Este estudo objetivou analisar fatores individuais, socioeconômicos, demográficos e de cobertura dos atendimentos não presenciais avaliados pelo CRIE Minas Gerais e identificar os determinantes que podem influenciar o acesso aos imunobiológicos especiais. A pesquisa busca aprimorar a compreensão dos fatores que podem afetar esse acesso, especialmente em contextos de desigualdade social, e reforça a relevância do CRIE Minas Gerais na promoção da equidade em saúde no estado. 

## Métodos 

### Desenho do estudo

Tratou-se de estudo epidemiológico, com delineamento misto, que utilizou dados referentes às solicitações de atendimentos virtuais do CRIE de Belo Horizonte, Minas Gerais, Brasil, no período de setembro de 2022 a julho de 2024.

### Contexto 

Até 2013, as solicitações de atendimentos de CRIE Virtual pelo CRIE Minas Gerais eram registradas por meio do Sistema de Informação dos Centros de Referência para Imunobiológicos Especiais (SI-CRIE), uma plataforma informatizada voltada ao gerenciamento de dados dos usuários e dos imunobiológicos especiais (1,3). Com o aumento da demanda e do volume de solicitações, o SI-CRIE tornou-se insuficiente para atender de forma eficaz às necessidades de registro do serviço. Diante dessa limitação e com base em orientações mais recentes, os registros dos atendimentos de CRIE Virtual passaram a ser feitos por meio do Google Forms, com armazenamento automático em planilhas do Google Sheets, o que favoreceu a organização e otimizou o gerenciamento das informações. Esse novo formato permite o registro detalhado dos usuários, agendamento de esquemas vacinais e monitoramento das vacinas liberadas, o que garante maior controle e eficiência das informações pelo CRIE Minas Gerais.

O CRIE Minas Gerais, enquanto CRIE Virtual, recebe solicitações de imunobiológicos especiais enviadas pelas unidades regionais de saúde, analisa-as e, caso confirme a indicação, autoriza e organiza a logística para que o imunobiológico seja ofertado nas macrorregionais do estado que não possuem um CRIE macrorregional (1,3). Essa estratégia também é adotada pelos demais CRIE da rede, otimizando os fluxos operacionais nas macrorregionais de saúde e ampliando a capacidade de resposta às solicitações recebidas pelos CRIE (4). As 28 gerências regionais de saúde preenchem as fichas de solicitação de imunobiológicos, elaboradas conforme os critérios do manual mais recente do CRIE, e as enviam virtualmente ao CRIE Minas Gerais.

Todas as solicitações recebidas e avaliadas pela equipe do CRIE Minas Gerais são registradas na planilha do Google Forms. Quando o usuário tem indicação para receber o imunobiológico, a solicitação retorna à gerência regional responsável, que repassa a demanda para a secretaria municipal de saúde. A vacinação do usuário ocorre na central de vacina ou na atenção primária, conforme a realidade do município. O serviço que administra a vacina é responsável pelo registro nos sistemas de imunização vigentes.

### Participantes 

Os participantes deste estudo foram os usuários do CRIE Minas Gerais com solicitações registradas nas planilhas do CRIE Virtual entre setembro de 2022 e julho de 2024. Foram excluídos os encaminhamentos em que não foi possível identificar corretamente o município de residência do usuário. Algumas variáveis apresentaram registros incorretos ou inválidos, reduzindo o número de registros conforme a variável analisada. Para a variável “indicação do imunobiológico”, foi feita uma categorização conforme as indicações do manual do CRIE vigente (3). A categoria “outras indicações” agrupou aquelas com menos de 1,0% de ocorrência na amostra estudada.

### Variáveis 

As variáveis utilizadas neste estudo contemplaram o perfil sociodemográfico do usuário, os dados do serviço de saúde encaminhador e as informações específicas da solicitação de imunobiológicos. Como variáveis independentes, foram adotadas as variáveis sociodemográficas dos municípios através do índice mineiro de responsabilidade social (12,13) de 2021, da Fundação João Pinheiro, e de índices de 2024 do Instituto Brasileiro de Geografia e Estatística, disponibilizados em sites de domínio público (https://imrs.fjp.mg.gov.br/Consultas e https://www.ibge.gov.br/estatisticas/sociais/populacao/9103-estimativas-de-populacao.html, respectivamente).

### Fontes de dados/mensuração 

Os dados foram obtidos a partir da extração das informações registradas nas planilhas oriundas das fichas de solicitação de imunobiológicos especiais recebidas pelo CRIE Minas Gerais, destinadas ao atendimento de CRIE Virtual. 

### Viés 

Com o intuito de evitar possíveis fontes de viés, após o recebimento da planilha, realizou-se a análise de consistência dos registros, que identificou dados incorretos ou faltantes lançados na variável “município”, impossibilitando sua inclusão no estudo. Dessa forma, foram excluídas 742 fichas das análises deste estudo.

### Tamanho do estudo

O estudo compreendeu todas as solicitações registradas entre setembro de 2022 e julho de 2024, considerando a totalidade dos atendimentos não presenciais avaliados pelo CRIE Minas Gerais. 

### Variáveis quantitativas

As variáveis do perfil sociodemográfico foram “sexo” (masculino/feminino) e “idade” (numérica e categórica). Para caracterizar o serviço encaminhador, utilizou-se a gerência regional de saúde (28 categorias). As informações da solicitação de imunobiológicos foram descritas pelas indicações/motivos dos encaminhamentos ao CRIE (15 categorias) e pelos imunobiológicos prescritos (19 categorias). As variáveis sociodemográficas dos municípios (famílias cadastradas com até meio salário mínimo; percentual de população pobre no CadÚnico; proporção da população atendida pela Estratégia de Saúde da Família; taxa de urbanização do município e população estimada do município) (12,13) foram conceituadas na Tabela 1.

**Tabela 1:** Variáveis de ambiente social utilizadas na regressão logística do estudo, com base no município de residência do indivíduo. Belo Horizonte, 2022-2024

Variáveis	Conceito
Famílias cadastradas com até meio salário mínimo	Quantitativo de famílias cadastradas com renda per capita de até meio salário mínimo que possuem até dois anos desde a data de sua inclusão ou última atualização cadastral.
Percentual de população pobre no CadÚnico	Proporção da população cadastrada no CadÚnico classificada como pobre, de acordo com critérios de renda estabelecidos pelo governo.
Proporção da população atendida pela Estratégia Saúde da Família	Percentual da população coberta pela Estratégia Saúde da Família, em determinado espaço geográfico, no ano considerado.
Taxa de urbanização do município	Percentual da população que vive em áreas urbanas em relação à população total do município, com base em dados do Instituto Brasileiro de Geografia e Estatística (IBGE).
População estimada do município	IBGE, Diretoria de Pesquisas, Coordenação de População e Indicadores Sociais: estimativas da população residente no Brasil e unidades da Federação com data de referência em 1º de julho de 2024.

### Métodos estatísticos

Os dados foram analisados com o programa estatístico Stata, versão 17.0. As variáveis categóricas foram descritas por frequências absolutas e relativas, e as variáveis contínuas, após verificação da assimetria dos dados, foram apresentadas por mediana e por intervalo interquartílico (IIQ). Para estimar a magnitude das associações entre a prescrição de imunobiológicos, o perfil sociodemográfico dos usuários e o município de origem, calculou-se a razão de chances (*odds ratio*, OR), brutas e ajustadas, com seus respectivos intervalos de confiança de 95% por meio de modelo de regressão logística.

Mapas coropléticos foram elaborados com o programa QGIS, versão 3.40.0, para representar a distribuição espacial dos encaminhamentos realizados no período do estudo.

## Resultados 

Durante o período do estudo, o CRIE Minas Gerais recebeu, na modalidade virtual, 9.900 fichas de solicitação de imunobiológicos. Após a aplicação dos critérios de exclusão, foram incluídas na análise 9.158 fichas. A maioria dos indivíduos solicitantes de imunobiológicos especiais era do sexo feminino (51,8%). A mediana de idade foi 51 anos (IIQ 26-77), com predominância da faixa etária de adultos, que representou 3.681 (40,2%) dos casos (Tabela 2). As indicações mais prevalentes para o recebimento de imunobiológicos foram: pessoas vivendo com HIV/aids (,2%) e doença pulmonar crônica, pneumopatias, asma (,1%) (Tabela 2). Entre os dados relacionados ao serviço de saúde encaminhador, destaca-se a gerência regional de saúde de Coronel Fabriciano, responsável por 13,4% dos encaminhamentos (Figura 2).

**Tabela 2:** Sexo, faixa etária e indicações de imunobiológicos especiais dos usuários encaminhados para atendimento não presencial no Centro de Referência para Imunobiológicos Especiais Minas Gerais. Belo Horizonte, 2022-2024

Variáveis sociodemográficas^a^	n (%)
Sexo	
Feminino	4.736 (51,8)
Masculino	4.402 (48,2)
**Faixa etária** (anos)	
0-9	1.699 (18,6)
10-18	210 (2,3)
19-59	3.681 (40,2)
>60	3.553 (38,9)
**Indicações/motivos dos encaminhamentos** (9.158)	
Pessoas vivendo com HIV/aids	1.481 (16,2)
Doença pulmonar crônica, pneumopatias, asma	1.197 (13,1)
Imunocomprometidos/imunodeficiência devido à imunodepressão terapêutica/imunodeficiência adquirida e congênita	944 (10,3)
Neoplasias	787 (8,6)
Sem critério/sem doença de base/aguardando documentação	783 (8,5)
Diabetes	758 (8,3)
Recém-nascido suscetível - prematuridade	537 (5,9)
Doença cardiovascular crônica	531 (5,8)
Asplenia anatômica ou funcional, hemoglobinopatias, doenças de depósito e outras condições associadas à disfunção esplênica	452 (4,9)
Transplantes (órgãos sólidos/medula/doadores)	436 (4,8)
Nefropatia crônica/síndrome nefrótica	352 (3,8)
Doença neurológica crônica incapacitante/doença convulsiva crônica/encefalopatias	336 (3,6)
Outras indicações^b^	210 (2,3)
Hepatopatias	200 (2,2)
Síndromes genéticas	154 (1,7)

^a^Os totais por variável podem variar em razão de registros incompletos. ^b^Outras indicações: evento adverso prévio/outros/implante coclear fístula liquórica e derivação ventrículo-peritoneal/profilaxia pós-exposição de risco/abuso sexual/comunicantes domiciliares de HBS/recém-nascido da mãe HBSAg positivo/doenças reumatológicas/doenças dermatológicas crônicas graves/profissional de saúde/acidente percutâneo/contato mucosa caso índice Ag HBS (+) ou de alto risco/gestantes/púrpura (asplenia/imunossupressão)/comunicantes sexuais/doença de depósito.

**Figura 2:** Gerências regionais de saúde que encaminharam formulários de solicitação de imunobiológicos especiais para atendimento não presencial no Centro de Referência para Imunobiológicos Especiais. Minas Gerais, 2022-2024 (n=9.158)

Dos 9.158 registros, a prescrição e a liberação de imunobiológicos foram efetivadas para 8.211 indivíduos (89,6%). Ressalta-se que um mesmo indivíduo pode ter apresentado indicação para mais de um imunobiológico, totalizando 28.588 doses prescritas. As vacinas mais frequentemente indicadas foram: pneumocócica 23-valente (7.098, 24,8%), *Haemophilus influenzae b* (4.019, 14,1%) e hepatite A (3.885, 13,6%) (Tabela 3).

**Tabela 3:** Imunobiológicos prescritos para usuários encaminhados para atendimento não presencial no Centro de Referência para Imunobiológicos Especiais Minas Gerais. Belo Horizonte, 2022-2024 (n=28.588)

Imunobiológico prescrito	n (%)
Pneumococo 23 valente	7.098 (24,8)
*Haemophilus influenzae* B	4.019 (14,1)
Hepatite A	3.885 (13,6)
Meningocócica C conjugada	3.106 (10,9)
Pneumocócica conjugada 13 V	2.684 (9,4)
Influenza	1.708 (6,0)
HPV4^a^	1.266 (4,4)
Meningocócica conjugada ACWY	1.041 (3,6)
Hepatite B	998 (3,5)
DTP acelular^b^	937 (3,3)
Hexa acelular (DTPa/VIP/Hib/HB)^c^	542 (1,9)
dTadulto^d^	465 (1,6)
Varicela	447 (1,6)
Pólio inativada	182 (0,6)
Triviral	116 (0,4)
Febre amarela	66 (0,2)
Imunoglobulina anti-hepatite-B	23 (0,1)
Imunoglobulina antitetânica	3 (0,0)
Imunoglobulina antirrábica	2 (0,0)
Total	**28.588** (100,0)

^a^Vacina papilomavírus humano 6, 11, 16 e 18 (recombinante); ^b^Vacina adsorvida difteria, tétano e pertussis (acelular); ^c^Difteria, tétano, coqueluche, hemófilos influenza B, poliomielite, hepatite B; ^d^Difteria e tétano adulto.

Para estimar a magnitude da associação entre as variáveis individuais e as do ambiente social com a prescrição de imunobiológicos, a análise ajustada demonstrou que, quanto maior a idade, maior foi a chance de prescrição. O sexo feminino também apresentou maior chance de prescrição em comparação ao masculino, com significância estatística. Em relação às variáveis independentes relacionadas ao ambiente social dos municípios de residência dos indivíduos, observou-se que, quanto maior o número de famílias com renda de até meio salário mínimo, menor foi a chance de prescrição de imunobiológicos. Já municípios com maiores percentuais de população pobre cadastrada no CadÚnico e com maior taxa de urbanização apresentaram maiores chances de prescrição (p-valor<0,05) (Tabela 4).

**Tabela 4:** Razões de chances (*odds ratio*, OR) ajustadas e intervalos de confiança de 95% (IC95%) ajustados para prescrição de imunobiológicos, segundo variáveis individuais e de ambiente social. Belo Horizonte, 2022-2024

Variáveis	OR ajustada (IC95%)	p-valor
**Variáveis individuais**		
**Idade** (anos)	1,02 (1,01; 1,02)	<0,001
Sexo		<0,001
Feminino	referência	
Masculino	1,57 (1,36; 1,80)	
**Variáveis do ambiente social**		
Número de famílias com renda de meio salário mínimo	0,99 (0,99; 1,00)	0,050
Percentual da população pobre de acordo com o CadÚnico	1,01 (1,00; 1,03)	0,004
Proporção da população atendida pela Estratégia de Saúde da Família	0,99 (0,99; 1,00)	0,533
Taxa de urbanização	1,01 (1,00; 1,02)	0,026
População estimada	1,00 (0,99; 1,00)	0,255

## Discussão 

Os resultados deste estudo demonstraram uma possível associação entre a prescrição de imunobiológicos nos atendimentos não presenciais avaliados pelo CRIE Minas Gerais e as variáveis individuais e ambientais relacionadas à saúde. Esse achado reforça a relevância de garantir o acesso igualitário aos serviços de saúde como um compromisso essencial para o alcance dos objetivos de desenvolvimento sustentável.

Nesta pesquisa, a maioria dos indivíduos solicitantes de imunobiológicos especiais era do sexo feminino, com mediana de idade de 51 anos. Essa análise refletiu tendências demográficas e epidemiológicas atuais, em que o envelhecimento populacional, somado à maior expectativa de vida entre as mulheres, evidencia a necessidade de políticas públicas voltadas à promoção do envelhecimento saudável e da qualidade de vida em todas as fases da vida (14). No Brasil, em 2021, as doenças imunopreveníveis foram a segunda principal causa de morte entre aquelas evitáveis por intervenções do SUS entre mulheres de 5 a 74 anos (15).

Análise referente ao período 1998-2008 apontou que a diferença no acesso e na utilização dos serviços de saúde entre os gêneros permanecia significativa, com os homens apresentando um acesso sete pontos percentuais inferior ao das mulheres (16). Em 2023, dados do Ministério da Saúde indicaram um aumento nos atendimentos individuais a homens na Atenção Primária à Saúde em comparação com 2022. No entanto, os homens ainda representavam apenas 33,4% dos atendimentos realizados em nível nacional (11). Dentre os responsáveis pelos encaminhamentos dos atendimentos não presenciais ao CRIE Minas Gerais, destacaram-se as gerências regionais de saúde de Coronel Fabriciano e de Ponte Nova, localizadas, respectivamente, nas regiões do Vale do Aço e da Zona da Mata, Minas Gerais. Tal prevalência pode ser justificada pelo fato de que essas regiões, até o ano de 2024, não contavam com uma unidade física do CRIE, fator que contribuiu para a elevada demanda por atendimentos e fundamentou a recente implantação de um centro em Ipatinga, matriciado pela gerência regional de saúde de Coronel Fabriciano (4).

As solicitações de atendimentos do CRIE Virtual feitas por usuários dos municípios pertencentes à unidade regional de saúde de Uberaba são direcionadas ao CRIE Macrorregional de Uberaba. Já o CRIE Macrorregional de Uberlândia é responsável pelos municípios das unidades regionais de saúde de Uberlândia e de Ituiutaba. O CRIE de Juiz de Fora, por sua vez, atende aos municípios da respectiva unidade regional de saúde. Dessa forma, essas unidades podem não encaminhar solicitações de atendimentos virtuais ao CRIE Minas Gerais, o que pode explicar a ausência desses encaminhamentos durante o período analisado neste estudo.

Como parte dessa organização desde o final de 2022, a Secretaria de Estado de Saúde tem se mobilizado para estruturar fluxos e desenvolver competências com vistas à descentralização dos CRIE em Minas Gerais. A busca pela redução das desigualdades no acesso e na qualidade dos serviços por meio da equidade e da descentralização é um dos pilares fundamentais para garantir que a atenção à saúde seja adequada às diferentes necessidades da população (17,18). 

Quase todas as solicitações do CRIE Virtual avaliadas pelo CRIE Minas Gerais resultaram na prescrição de imunobiológicos especiais para os indivíduos encaminhados. A assertividade dos profissionais responsáveis pelos encaminhamentos reforça a importância do conhecimento técnico acerca dos grupos contemplados e das respectivas recomendações. As indicações, os esquemas vacinais diferenciados, os critérios preestabelecidos e os endereços dos CRIE estão disponíveis no Manual dos CRIE (3).

O domínio do fluxo, das indicações, das normas e dos procedimentos necessários para o encaminhamento de pessoas em situação de maior vulnerabilidade destaca a discussão sobre o acesso desses usuários aos serviços de saúde (5-10). Não foi possível verificar se os imunobiológicos prescritos foram efetivamente administrados, bem como o intervalo de tempo entre a solicitação, a liberação e a aplicação das doses. Tal limitação decorre da ausência de integração entre os registros analisados e os sistemas nacionais de informação em saúde, como o SI-PNI e o e-SUS, nos quais são registrados o recebimento e a aplicação dos imunobiológicos.

Dentre as indicações/motivos para os encaminhamentos à vacinação no CRIE, destacaram-se as condições de pessoas vivendo com HIV/aids e de doença pulmonar crônica, pneumopatias e asma. Neste estudo, a vacina pneumocócica 23-valente foi a mais frequentemente prescrita. A Pneumonia Adquirida na Comunidade foi identificada como a terceira coinfecção mais prevalente entre pessoas vivendo com HIV, em um estudo longitudinal (19). Esses achados reforçam que a infecção pelo HIV torna os indivíduos mais vulneráveis a infecções quando comparados à população geral (3). 

Uma metanálise demonstrou redução dos casos de pneumonia infantil causados por *Haemophilus influenzae* tipo B (Hib) após o licenciamento da vacina conjugada contra Hib na China, evidenciando que o uso ampliado dessa vacina contribuiu para a diminuição do impacto do patógeno entre os causadores de pneumonia infantil no país (20). A segunda vacina mais prescrita foi contra hepatite A. A incidência dessa infecção em populações de baixa renda ocorre em idades mais precoces, em razão da carência de saneamento básico. Por outro lado, em regiões com melhores condições sanitárias, observa-se maior incidência entre adolescentes e adultos (3). 

O CRIE também pode indicar vacinas não prescritas pelo médico assistente que encaminhou a solicitação, o que reforça a importância da atuação de uma equipe técnica composta por médico e por enfermeiro no CRIE Virtual (3,4). Após a análise das solicitações, esses profissionais são capazes de ampliar o acesso às vacinas disponibilizadas pelo serviço. Dessa forma, os atendimentos não presenciais avaliados pelo CRIE Minas Gerais podem, igualmente, ser influenciados por fatores contextuais, especialmente de natureza social.

Neste estudo, buscou-se investigar a existência de desigualdades ou de padrões sociais implícitos na prescrição de imunobiológicos especiais, ainda que essa prescrição devesse basear-se exclusivamente em critérios clínicos. Os resultados demonstraram que o aumento da idade esteve associado à maior chance de prescrição de imunobiológicos e que as mulheres apresentaram maior probabilidade de prescrição em comparação aos homens. Essa heterogeneidade entre homens e mulheres, bem como entre jovens e idosos, também foi observada em um estudo sobre a cobertura vacinal contra a covid-19 no Brasil, o qual identificou que as populações mais idosas foram priorizadas para vacinação e que, em todas as faixas etárias, a cobertura vacinal entre as mulheres manteve-se superior à observada entre os homens ao longo do tempo (21).

O modelo identificou que indivíduos residentes em municípios com maior taxa de urbanização apresentaram maior probabilidade de receber prescrição de imunobiológicos. Um estudo realizado na Etiópia, que comparou crianças totalmente vacinadas em áreas urbanas e em áreas rurais, constatou prevalência mais elevada de vacinação completa entre residentes em áreas urbanas (22).

Neste estudo, observou-se ainda que o número de famílias com renda de até meio salário mínimo associou-se à menor chance de prescrição, enquanto o maior percentual de população pobre cadastrada no CadÚnico relacionou-se à maior chance de prescrição. Assim, dois indicadores socioeconômicos mostraram possível associação com a probabilidade de o indivíduo receber imunobiológicos. Durante a pandemia, a renda também influenciou a continuidade do esquema vacinal: moradores de municípios com menor renda per capita apresentaram maior probabilidade de não receber a segunda dose da vacina contra a covid-19 (21).

Uma análise global sintetizou evidências de desigualdades socioeconômicas na aceitação das vacinas de rotina e mostrou associação positiva entre status socioeconômico elevado e adesão à vacinação na maioria dos estudos (23). Em contrapartida, inquérito sobre cobertura e hesitação vacinal nas capitais brasileiras revelou menor adesão à vacinação nos estratos mais favorecidos em comparação aos mais vulneráveis (24). Assim, uma distribuição equitativa de vacinas, em âmbito nacional e em internacional, é essencial para garantir o acesso universal à imunização e a redução das desigualdades em saúde (7-9,25,26).

Estudo na África Subsaariana identificou que crianças provenientes de famílias com maior renda apresentaram maiores taxas de cobertura vacinal em comparação àquelas de famílias mais pobres (27). Além das condições econômicas, aspectos relacionados ao acesso físico aos serviços de saúde, como proximidade das unidades básicas de saúde, horário de funcionamento e ocorrência de faltas temporárias de vacinas, também influenciaram significativamente a adesão (27). Destaca-se, ainda, o acesso à informação, especialmente por meio da recomendação dos profissionais de saúde, que tem impacto comprovado na decisão de se vacinar ou de vacinar seus dependentes (27).

Em 2025, com o objetivo de ampliar o acesso e otimizar os serviços ofertados pelos CRIE, o Ministério da Saúde instituiu, por meio de portaria, a criação da Rede de Imunobiológicos para Pessoas em Situações Especiais (RIE). Essa nova estratégia não substitui os CRIE, que permanecem responsáveis pela validação das indicações, pela administração de vacinas especiais e pela atuação como referência territorial pactuada nas comissões intergestores bipartite. A principal inovação trazida pela RIE é a ampliação da capilaridade de atendimento, permitindo que a Atenção Primária à Saúde, por meio das unidades básicas de saúde, também realize a vacinação de pessoas com condições especiais, favorecendo a integração dos níveis de atenção e promovendo maior equidade no acesso aos imunobiológicos (28).

Por fim, acredita-se que os resultados deste estudo fornecem subsídios importantes para o aprimoramento e a operacionalização dos CRIE, ao evidenciar potencialidades no processo de acesso aos imunobiológicos especiais disponíveis para a população em situação especial, considerando seu contexto social. Na prática, essas informações possibilitam o desenvolvimento de ações de planejamento, de monitoramento e de avaliação, com o objetivo de otimizar a efetivação da equidade no acesso a imunobiológicos especiais, bem como contribuir para a reorganização dos fluxos e para a ampliação da vacinação em populações vulneráveis.

Este estudo apresenta como limitação o uso de dados secundários, sujeitos a possíveis preenchimentos incorretos ou incompletos. Além disso, por se tratar de dados anonimizados, não foi possível identificar a presença do mesmo indivíduo em diferentes registros, critério necessário para garantir a independência das variáveis explicativas empregadas no modelo de regressão. Outro fator limitante foi a ausência de informações que permitissem distinguir a origem do serviço de saúde responsável pelo encaminhamento, se público ou privado, o que poderia enriquecer a análise das variáveis de contexto social. Ressalta-se, ainda, que não foi possível confirmar a administração nem o tempo entre a solicitação, a liberação e a aplicação do imunobiológico, devido à falta de integração dos sistemas de registro. Todavia, destaca-se a aplicação de metodologia rigorosa, que permitiu identificar o padrão aleatório dos dados faltantes e realizar análises de sensibilidade, para conferir confiabilidade aos resultados apresentados.

Houve potencial associação entre os determinantes ambientais da saúde e os atendimentos não presenciais avaliados pelo CRIE Minas Gerais. A identificação do perfil do ambiente social desses atendimentos em Minas Gerais suscita a discussão sobre a necessidade de garantir a equidade no acesso à saúde. Assim, assegurar que todos tenham acesso igualitário aos serviços de saúde é um compromisso ético e uma estratégia indispensável para alcançar os objetivos de desenvolvimento sustentável. A ampliação de pesquisas e de dados que mensurem o impacto de ações equitativas permite avaliar o progresso e propor intervenções mais eficazes no combate às desigualdades.

## Data Availability

Os dados utilizados neste estudo não podem ser disponibilizados publicamente em sua totalidade, em razão de impedimentos éticos estabelecidos pelo Comitê de Ética em Pesquisa que aprovou a pesquisa, sob o Certificado de Apresentação de Apreciação Ética nº 78079324.2.0000.5149 e Parecer nº 6.739.290. Dessa forma, os dados devidamente anonimizados foram depositados no repositório da SciELO Data e foram seguidas rigorosamente as exigências éticas quanto à confidencialidade e ao sigilo das informações.
